# Percutaneous Holmium Laser Fulguration of Calyceal Diverticula

**DOI:** 10.1155/2012/716786

**Published:** 2012-02-13

**Authors:** Amjad Alwaal, Raed A. Azhar, Sero Andonian

**Affiliations:** Division of Urology, McGill University Health Centre, Montreal, QC, Canada H3A 1A1

## Abstract

*Introduction*. Calyceal diverticular stones are uncommon findings that represent a challenge in their treatment, due to the technical difficulty in accessing the diverticulum, and the high risk of their recurrence. Current percutaneous technique for calyceal diverticular stones involves establishing a renal access, clearing the stone, and fulguration of the diverticular lining with a roller-ball cautery electrode using hypotonic irrigation solution such as sterile water or glycine solution which may be associated with the absorption of hypotonic fluids with its inherent electrolyte disturbances. *Case Report*. In this paper, we present for the first time percutaneous holmium laser fulguration of calyceal diverticula in 2 patients using normal saline. Their immediate postoperative sodium was unchanged and their follow-up imaging showed absence of stones. Both patients remain asymptomatic at 30 months post-operatively. *Conclusion*. This demonstrates that holmium laser is a safe alternative method to fulgurate the calyceal diverticulum after clearing the stone percutaneously.

## 1. Introduction

Calyceal diverticulum is a congenital thin-walled, urothelium-lined cavity that communicates with the collecting system through a narrow ostium. This narrow neck allows the diverticulum to be filled passively with urine. It is an uncommon finding that is found incidentally in 0.21–0.45% of individuals undergoing renal imaging [1, 2]. Stones complicate 9.5–50% of calyceal diverticula, resulting in pain and/or hematuria [1, 3]. 

Shockwave lithotripsy can produce symptomatic relief from diverticular stone. However, it is associated with a low stone-free rate of 21% [4]. Although retrograde ureteroscopic and laparoscopic approaches have been described, percutaneous management of calyceal diverticula represents the cornerstone in the management of calyceal diveritcular stone disease and offers the highest stone-free rate of 90% [5–9]. Current percutaneous techniques for calyceal diverticular stones involve establishing a renal access, clearing the stone, and fulgurating the diverticular lining with a roller-ball electrode cautery using a hypotonic irrigation solution such as sterile water or glycine [10]. However, this may be associated with absorption of hypotonic fluids with its inherent serum electrolyte disturbances. Therefore, the aim of the present study was to apply holmium:yttrium-aluminum-garnet (Ho:YAG) or the holmium laser technology for fulguration of calyceal diverticula in 2 patients using normal saline.

## 2. Case Presentation

### 2.1. Patient 1

**Figure 1:**
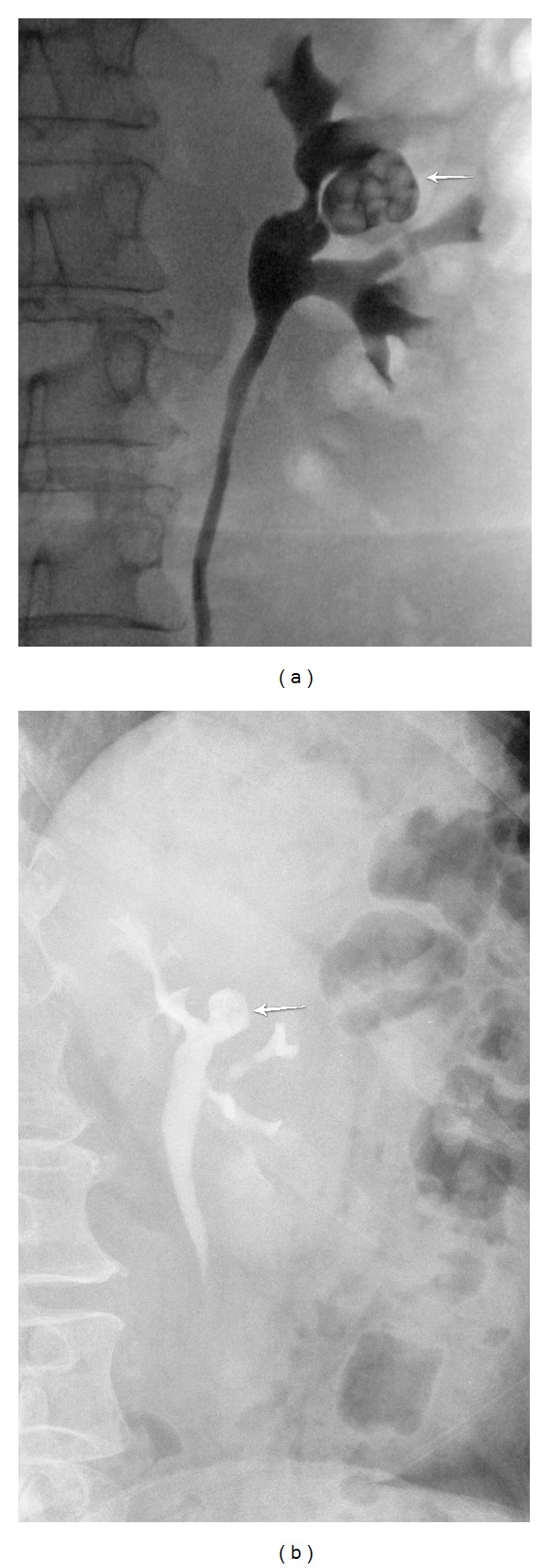
(a) Patient 1: preoperative left retrograde pyelogram demonstrating calyceal diverticulum containing stones. (b) Patient 1: 15-minute film of IVP at 11 months after percutaneous holmium laser fulguration of the calyceal diverticulum demonstrating absence of stones and stable size of the diverticulum.

A 56-year-old woman presented with flank pain without significant past medical history (Table 1). IVP showed the presence of a 2 × 2.1 cm calyceal diverticuum in the anterior aspect of the left upper pole (Figure 1(a)). The IVP also showed the presence of a mild degree of bilateral medullary-sponge kidney disease. *Pseudomonas aeruginosa* urinary tract infection was treated with a course of ciprofloxacin. A month prior, she had a failed attempt of percutaneous diverticulectomy by another urologist. She had a re-entry 20 F Malecot nephrostomy tube.

### 2.2. Patient 2

A 64-year-old woman presented with flank pain and frequent urinary tract infections (Table 1). Her past medical history was significant for hypothyroidism. A CT scan of the abdomen showed the presence of a mid-pole calyceal diverticulum on the anterior aspect containing 2 stones (1.8 × 1.1 cm and 1.4 × 1.4 cm) in a cavity of 2.4 × 1.4 cm. Urine culture grew *Klebsiella*, which was treated with ciprofloxacin.

### 2.3. Technique

**Figure 2:**
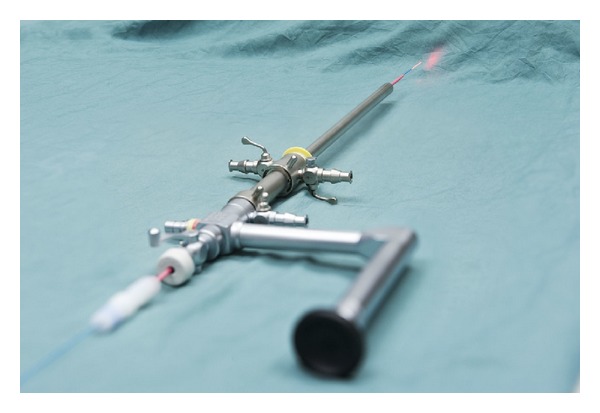
A photograph of the nephroscope setup: a 365 *μ* holmium laser fiber (SlimLine 365 micron Blue Jacket Reusable Fiber; Lumenis Inc., Santa Clara, CA) stabilized with a 7 F ureteral catheter (Cook, Bloomington, IN) and used through the indirect nephroscope.

For the first patient, an indwelling 16 F Foley catheter was inserted into the bladder and the patient was positioned into prone position. Access into the calyceal diverticulum was gained through the previous nephrostomy access. For the second patient, flexible cystoscopy was performed and a 5 F ureteral catheter was placed into the renal pelvis under fluoroscopic guidance. The ureteral catheter was secured to the 16 F Foley catheter and the patient was positioned into prone position. Using injection of mixture of contrast dye and indigo carmine, access into the calyceal diverticulum was obtained with a diamond-tipped 18 G needle. This required 2 punctures. After placing guidewires, the tract was dilated using the X-Force N30 balloon dilator (Bard, Covington, GA). Both patients had only one percutaneous tract. A 30 F Amplatz sheath and an indirect nephroscope were used to visualize the stones. Stones were fragmented and aspirated with the Swiss LithoClast Ultra (Boston Scientific, Natick, MA). Stone-free status was confirmed by both fluoroscopy and direct visualization using a flexible nephroscope. For the first patient, the calyceal ostium was identified and dilated to 20 F using Amplatz dilators. For the second patient, the calyceal ostium was not identified. Once stone-free, a 365 *μ* holmium laser fiber (SlimLine 365 micron Blue Jacket Reusable Fiber; Lumenis Inc., Santa Clara, CA) stabilized with a 7 F ureteral catheter (Cook, Bloomington, IN) was used through the indirect nephroscope to fulgurate the calyceal diverticular mucosa at 10 watts energy (1J × 10 Hz) (Figure 2). A 100 W Ho:YAG laser generator (VersaPulse PowerSuite; Lumenis Inc., Santa Clara, CA) was used. Normal saline irrigation solution was used during the lithotripsy and laser fulguration of the mucosa. Once all of the mucosa was fulgurated and hemostasis was insured, the procedure was terminated. For the first patient, an antegrade 6 F × 30 cm double pigtail stent was placed with the proximal coil in the calyceal diverticulum and the distal coil in the bladder. The skin was closed in a subcuticular fashion with an absorbable suture. For the second patient, an 8.5 F cope-loop nephrostomy tube was placed into the calyceal diverticulum. Intraoperatively, both patients received 1.3 g of acetaminophen rectally and ketorolac 30 mg intravenously.

Postoperatively, both patients had acceptable chest X-rays and hematocrits. Postoperative serum sodium remained stable (Table 1). Furthermore, since pain was well controlled with oral narcotics, both patients were discharged home on the same day with home care nurse for dressing changes. The first patient was discharged home with Foley catheter that was removed 2 days later in the office. The double pigtail stent was removed cystoscopically a month later. The second patient was discharged home the same day with the nephrostomy tube, which was subsequently removed 9 days later after a nephrostogram confirming stone-free status and good drainage of the calyceal diverticulum. There were no intraoperative or postoperative complications in both patients. For the first patient, IVP performed at 2, 11, and 24 months postoperatively showed absence of stones and significant reduction in the size of the calyceal diverticulum (Figure 1(b)). A postoperative IVP at 12 months in the second patient showed significant reduction of the calyceal diverticulum without evidence of stones. Both patients remain asymptomatic at 30 months.

## 3. Discussion

Minimally invasive treatment options for calyceal diverticula include percutaneous surgery, ureteroscopy, and laparoscopic surgery. Most investigators agree that eradication of the calyceal diverticula is essential for the prevention of stone recurrence in these patients. The percutaneous approach has a high stone-free rate of 90% [9]. Traditionally, this approach required dilatation of the ostium and fulguration of the mucosa. Recently, Kim et al. have demonstrated that dilatation of the tract is not necessary. Their technique involved the fulguration of the diverticulum using a roller-ball electrode without cannulating or dilating the infundibulum [10]. However, this is done using hypotonic irrigation solution thus the potential risk of postoperative serum electrolyte disturbances.

Holmium:yttrium-aluminum-garnet (Ho:YAG) or the holmium laser has a wavelength of 2100 nm, which is absorbed by water [11]. Furthermore, its depth of penetration is only 0.5 to 1 mm [12]. In addition, holmium laser provides the advantage of using normal saline as an irrigation solution. In a prospective study of holmium laser enucleation of the prostate, it was found that the procedure was not associated with dilutional hyponatremia, and it did not affect the sodium concentration postoperatively [13]. Therefore, it is safer to use holmium laser and it allows for a longer operative time. In this initial case report, we present for the first time percutaneous Holmium laser fulguration of calyceal diverticula in 2 patients using normal saline. In the present study, the postoperative serum sodium remained stable or increased (Table 1). Furthermore, the procedures were performed in an ambulatory setting since both patients were healthy with satisfactory post-operative chest X-rays and hematocrits (Table 1). Follow-up IVP indicated stone-free status at 24 months in the first patient and at 12 months in the second patient; both were symptom-free at 30 months. However, larger sample size is required to confirm these results.

A controversy exists whether underlying urinary metabolic abnormalities in patients with calyceal diverticular stones exist [14–16]. In the present study, both patients showed metabolic stone work-up abnormalities that were treated adequately (Table 1).

## 4. Conclusion

Holmium laser is a safe and effective alternative method of fulgurating calyceal diverticular mucosa after clearing calyceal stones percutaneously. A limitation of the study is that it is an initial report, and a longer followup with a larger patient population is needed.

## Figures and Tables

**Table 1:** Patient characteristics.

Characteristics	Patient 1	Patient 2
Age and sex	56 F	64 F
ASA	1	2
BMI	25	23
Side	Left	Right
Location	Upper pole, anterior	Midpole, anterior
Stone size	2 cm × 2.1 cm	1.8 cm × 1.1 cm and 1.4 cm × 1.4 cm
Hounsfield units	1034	1294
Preop serum sodium	138 mEq/L	142 mEq/L
Postop serum sodium	142 mEq/L	142 mEq/L
OR time	85 min	95 min
Fluoroscopy time	5 min	4 : 45 min
Postop Hct	0.36	0.43
PACU stay	5 hours	3 hours
PACU narcotics (mg morphine equivalents)	93 mg	50 mg
Stone composition	60% calcium phosphate	100% calcium phosphate
	30% calcium oxalate monohydrate	
	10% calcium oxalate dihydrate	
Metabolic stone workup	pH 5.5, hypercalciuria, hyperuricosuria, hypernatriuria, and hypocitraturia	pH 7, hypercitraturia
Longterm prophylaxis	Low salt and purine diet; allopurinol, potassium citrate, and hydrochlorothiazide	trimethoprim/sulfamethoxazole

## Abbreviations

ASA: American Society of Anesthesiology
KUB: Kidney Ureter Bladder Film
IVP: Intravenous pyelogram.
